# Speech impairment detection in children using time frequency features of speech and deep learning techniques

**DOI:** 10.3389/fnhum.2026.1766439

**Published:** 2026-05-29

**Authors:** Manisa Manoswini, Brijesh Raj Swain, Biswajit Sahoo, Mahendra Kumar Gourisaria, Amitkumar V. Jha, Nicu Bizon, Aleena Swetapadma

**Affiliations:** 1 School of Computer Engineering, KIIT Deemed to be University, Bhubaneswar, India; 2 P.G. Department of Medicine, Sum Hospital, SOA University, Bhubaneshwar, India; 3 School of Electronics Engineering, KIIT Deemed to be University, Bhubaneswar, India; 4 The National University of Science and Technology POLITEHNICA Bucharest, Piteşti University Centre, Pitesti, Romania

**Keywords:** artificial intelligence, CNN, DWT, GFCC, GRU, LSTM, shallow learning, speech impairment

## Abstract

Speech impairment in children is nowadays occurring more commonly than in earlier days. It is necessary to detect the impairment in speech as early as possible to provide therapeutic treatments. In this work, a novel method has been suggested for speech impairment detection. First, speech signals are collected from children, including vowels, consonants, and different syllables. The speech signals are then processed using gammatone filter bank-based cepstral coefficients (GFCC) and discrete wavelet transform (DWT) for time–frequency feature extraction. DWT is a time-frequency feature that is extracted with the Haar wavelet. The proposed work uses a 5-fold cross-validation method for implementation. The features are then given to various learning technologies for designing the training speech impairment detection module. The learning technologies used are shallow learning (multilayer perceptron (MLP) and support vector machine (SVM)), shallow ensemble learning (gradient boosting (GBoost), extreme gradient boosting (XGBoost), categorical boosting (CatBoost), light gradient boosting machine (lightGBM), Stacking) and deep learning (Convolutional long short term memory (ConvLSTMs), bidirectional LSTM (BiLSTMs), convolutional neural network LSTM (CNN-LSTM), gated recurrent units CNN (GRU-CNN)) methods. The training module is then tested using speech signals that were not given in training. The test results of the methods have been analysed to select an optimal method. It is observed that the highest accuracy of 99.67% is obtained using the GRU-CNN method with DWT features. The contribution of the proposed method is that the DWT features with the GRU-CNN model have not been suggested for speech impairment detection until now with this accuracy. Yet another contribution is that all speech signals combined are given as input in one module, unlike existing methods that are designed with a separate module for each type of speech. Based on the findings, it is feasible to develop a computational framework for detecting speech impairment based on time frequency representations of speech signals along with deep learning methods.

## 1 Introduction

Effective speech communication depends on both verbal and non-verbal elements, such as speech clarity and body language, which together influence how individuals are perceived and how they respond (Spence, 2003). Speech is a fundamental element of human communication and plays a crucial role in logical and social development, particularly in children. Speech impairments affect the pronunciation, articulation, fluency, and phonation of spoken sounds, which may effect communication effectiveness. Early detection of speech impairment is important because timely therapeutic intervention can significantly improve communication outcomes. Computational methods have the potential to provide efficient tools for early screening of speech disorders by automatically analysing acoustic characteristics of speech signals. Machine learning (ML) and deep learning (DL) approaches have shown good results in detecting problems in speech production by identifying patterns in time-frequency representations of audio signals (Zhang et al., 2025). These approaches can potentially support clinicians by improving diagnostic accuracy and reducing manual effort (Remya et al., 2025). Speech impairment affects 2 to 7% of children, with nearly 50–70% having a history of genetic inheritance (Kaushik et al., 2021; Tomblin et al., 1997). Early detection is vital, and using computational methods can save time and give more accurate results compared to traditional evaluation processes. Deep learning has shown tremendous advantages in various applications (Ilie et al., 2025). Recent advancements like DL and ML help in the identification of speech problems, which is more cost-effective. Advanced technologies like neighbourhood component analysis (NCA) and a weighted k-NN classifier were used to achieve 97.93% accuracy for speech classification (Sharma and Singh, 2020b). A Naive Bayes classifier demonstrated LPC’s potential for identifying neuro-developmental problems by achieving 97.9% accuracy with the peak 20 LPC features and 97.8% accuracy with the peak 10 features by utilising LPCs’ capacity to simulate the human voice system (Sharma and Singh, 2020a). A machine learning-based approach that identified speech impairment in toddlers with 99% accuracy, 98.96% sensitivity, and 99.20% specificity used spectrogram-derived textural features, namely 14-dimensional Haralick’s features and 59-dimensional local binary patterns (LBPs) (Sharma G. et al., 2020). Audio texture and age-wise analysis of disordered speech in children have also been discussed (Sharma G. et al., 2021).

A hybrid CNN-LSTM model and a customized CNN were used in a recent study to detect speech impairment with 100% accuracy. By contrast, the average accuracy of the standalone CNN was 90% (Sharma and Singh, 2021). The Sch-Net neural network, which was improved by incorporating skip connections and a convolutional block attention module (CAM), reported impressive results, achieving 97.68% accuracy for identifying schizophrenia speech and 99.52% accuracy for distinguishing speech impairment (Fu et al., 2021). Additionally, a thorough machine learning framework was presented that included statistical and textural descriptors, SVM classification, wavelet packet decomposition (WPD), and feature extraction based on the molecular structure of favipiravir (Barua et al., 2023). Improved conditional random fields have also been used for speech impairment detection (Deepa, 2023). Recent research has incorporated a speaker-independent approach for identifying speech impairment in children using MFCC features combined with neural networks and linear prediction coefficients (LPC) with multilayer perceptron (MLP). This method achieves impressive accuracy and efficiency in classifying speech impairment while utilizing a minimal set of features, out performing current methods (Safdar et al., 2023). In another study, the interpretable multi-scale feature extraction model for enhancing automatic pathological voice detection through transparent feature extraction and comprehensive result evaluation has been proposed (Zhao et al., 2024). It improves accuracy up to 0.1977 and Matthews correlation coefficients (MCC) up to 0.4463. A deep learning model was designed to classify speech disorders using MobileNetV3 and EfficientNetB7 for feature extraction, integrated with the CatBoost classifier (Dutta and Wahab Sait, 2024). Table 1 noted all the methods and features used for Speech impairment detection. Some methods use time–frequency domain parameters along with SVM and feed-forward neural networks to detect speech impairment (Reddy et al., 2020). Some methods combine MFCC and i-vectors as a feature vector to identify speech impairment using pre-trained deep learning models (Kasture and Jain, 2024). Chroma, MFCC, and Tonnetz features along with SVM, random forest and recurrent neural network, have been suggested for speech impairment detection (Slogrove and van der Haar, 2020). MFCC and random forest have an accuracy level of 99%.

**Table 1:** Existing studies on speech impairment detection using acoustic features.

Authors	Features used	Method used
Kaushik et al. (2021)	STFT spectrograms	CNN
Sharma and Singh (2020b)	Pitch-based statistical features	k-NN classifier
Sharma and Singh (2020b)	Linear predictive coding coefficients	Naive-Bayes and SVM
Sharma G. et al. (2020)	Spectrogram texture anlysis using Harslick and local binary pattern features	SVM
Sharma G. et al. (2021)	LBP and EMS features	SVM, k-NN, Complex tree
Sharma and Singh (2021)	CNN features	Customized CNN and Hybrid CNN-LSTM models
Fu et al. (2021)	Converted to spectrograms using Short Time Fourier Transform (STFT)	Sch-net (CNN with skip connections and CBAM)
Barua et al. (2023)	Favipiravir pattern	WPD, INCA, and SVM classifier
Deepa (2023)	Common Voice Tool and XLS-R Fine Tuner for audio-to-text corpus translation.	Improved Conditional Random Fields
Safdar et al. (2023)	MFCC and LPC	Neural Network and MLP
Zhao et al. (2024)	AT-SincNet and interpretable feature extraction	IMBFN: Depth-wise separable CNN
Dutta and Wahab Sait (2024)	MobileNet V3, EfficientNet B7	CatBoost

Furthermore, several existing methodologies require designing separate modules for each type of speech input. Although these studies often report high accuracy, they rarely validate their methods on large-scale datasets. In addition, the commonly used MFCC feature extraction technique is highly sensitive to noise, which can significantly degrade model performance. A new method that uses the discrete wavelet transform (DWT) for feature extraction has been put forth to get over these restrictions and provide a more reliable representation of speech signals. This all-inclusive framework is a useful tool for speech pathologists and other clinical practitioners since it attempts to enhance the identification of speech problems in children while retaining high accuracy. The following are the main contributions of the suggested speech impairment detection study:

- For automatic speech impairment classification, a hybrid GRU-CNN architecture is presented that combines convolutional and recurrent learning to efficiently capture both local and temporal aspects of speech.
- When combined with deep learning models, the discrete wavelet transform (DWT) for feature extraction performs better than conventional GFCC-based features in terms of accuracy and generalisation.
- Using the entire LANNA dataset, which includes vowels, consonants, syllables, and challenging words provides a more accurate evaluation of speech than previous research.
- It is benchmarked over 15 models, including traditional machine learning and deep learning approaches, providing a broad evaluation and highlighting the superiority of the proposed GRU-CNN with DWT.
- It achieved state-of-the-art performance, with 99.67% accuracy, along with perfect precision, recall, F1-score, and AUC. This highlights the model’s clinical relevance for real-time screening and diagnostic support in speech-language pathology.

## 2 Materials and methods

**Figure 1:**
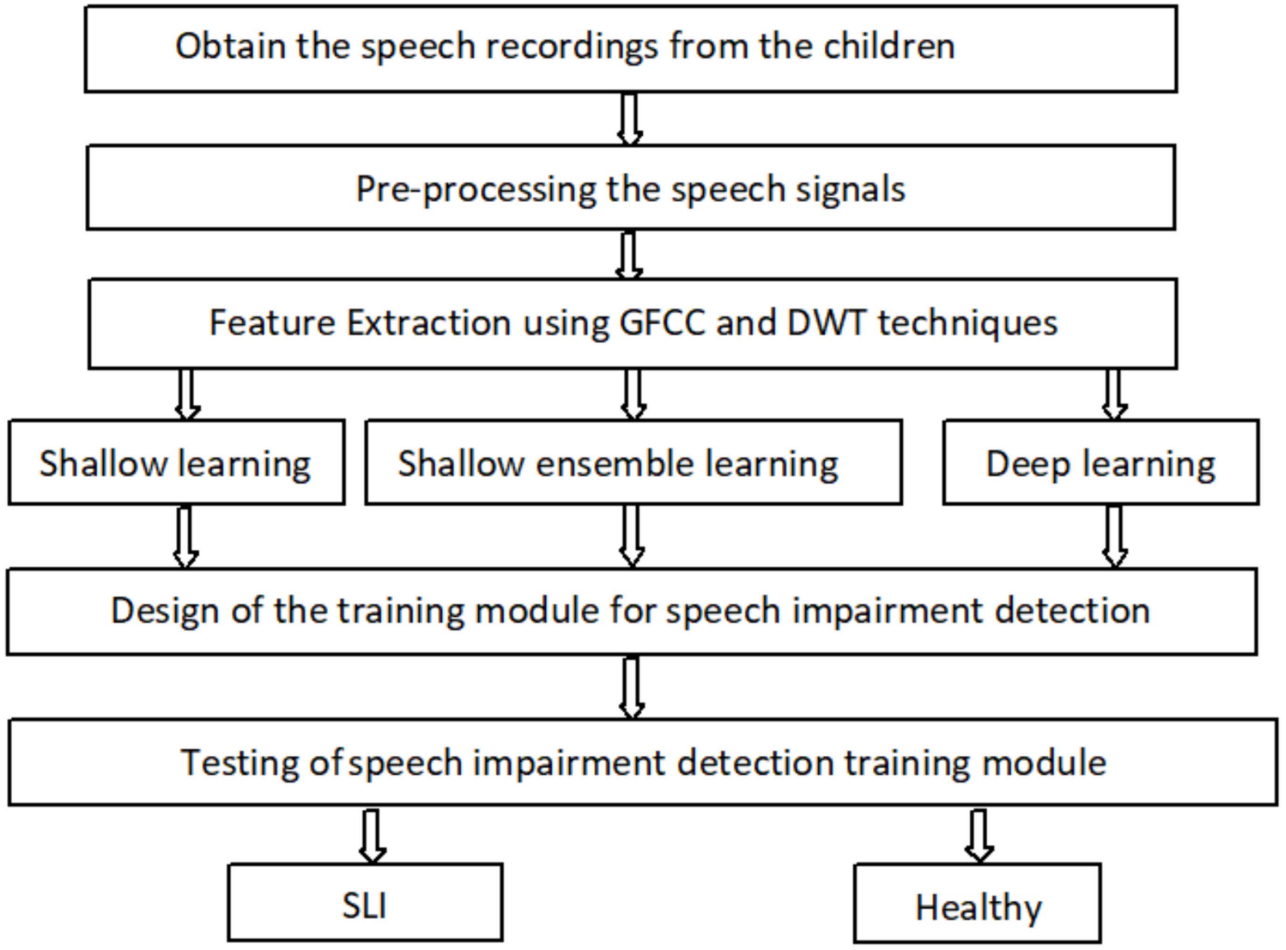
Proposed method for speech impairment detection.

The novel approach for speech impairment detection consists of various steps, as shown in Figure 1. This process starts with recording the speech of 2 to 5-year-olds, which are recorded individually. These speech recordings are the primary input for this analysis. Then the recordings are pre-processed, where it enhanced the quality of speech using various techniques, such as noise reduction and normalization so that it will be more effective while feature extraction. The pre-processed speech then employs feature extraction method. This technique analyzes the speech signals to extract relevant features that characterize the speech, making it easier to differentiate between healthy speech and affected speech. When the features are extracted then these features are utilized in the both training and testing phase, where classifiers learn to recognize the patterns and then conduct the evaluation of the performance of the data set separately. The last output of this process is the classification step. Here, the trained classifiers categorize the speech recordings into impaired and healthy by providing valuable information for the early detection and appropriate management of speech disorders.

### 2.1 Inputs used

To validate the proposed method, LANNA speech data-set has been used (Rudzicz et al., 2012). This data-set contains voice recordings from children aged 4–12 years, with both healthy individuals and speech impairment individuals. Those recordings contain vowels, consonants, one-syllables to four syllables and difficult words. It consists of 98 children, with 44 normal children and 54 children diagnosed with speech impairment. The 3,844 recordings include 1952 from patients and 1892 from healthy individuals. Some challenging sounds are taken to check the difficulties of children so that it will allow the researchers to analyze the patterns for early diagnosis and management of speech impairments. Voice clips were recorded and then preprocessed before feature extraction, which involved storing the clips in WAV format. Each audio clip was first loaded through librosa while retaining its original sampling rate. Audio clips were normalized to minimize differences in their amplitude values. Only valid clips were retained after preprocessing. All recordings are stored in WAV format to get accurate classification between healthy and speech impairment individuals for research in pathological voice detection in early diagnosis challenges and speech therapy.

### 2.2 Techniques used

Artificial intelligence has been used in health care in the last few decades (Danescu et al., 2025). AI methods most commonly used are classification methods for identifying the class (Doru and Costel, 2023). The techniques used are basically the signal processing techniques and classification techniques. The signal processing techniques used for processing the speech signals are GFCC and DWT. The classification techniques used are shallow learning methods, shallow ensemble learning methods and deep learning methods. The shallow learning methods used are SVM and MLP. The ensemble shallow learning methods used are RF, GBoosting, XGBoosting, CatBoosting, lightGBM and stacking. The deep learning methods used are 1D Conv, ConvLSTM, BiLSTM, DBN, and GRU-CNN. All the methods have been discussed in the section below in detail.

#### 2.2.1 GFCC

The feature extraction process involves segmenting speech into small intervals referred to as frames. Variability among different speakers can adversely affect the speech signal, influenced by factors such as channel effects and background noise. In the proposed study, the gammatone filter bank-based cepstral coefficients (GFCC) method is utilized, which is based on the gammatone filter bank. Mel-frequency cepstral coefficients (MFCC) and GFCC extraction are quite similar, which emphasizes how well GFCC extracts important speech characteristics. GFCC is a technique used in speaker identification systems that employs FFT. It provides better resolution at lower frequencies and performs better in noisy environments compared to MFCC due to its use of cube root transformation instead of logarithmic scaling (Arora and Neeru, 2016).

**Figure 2:**
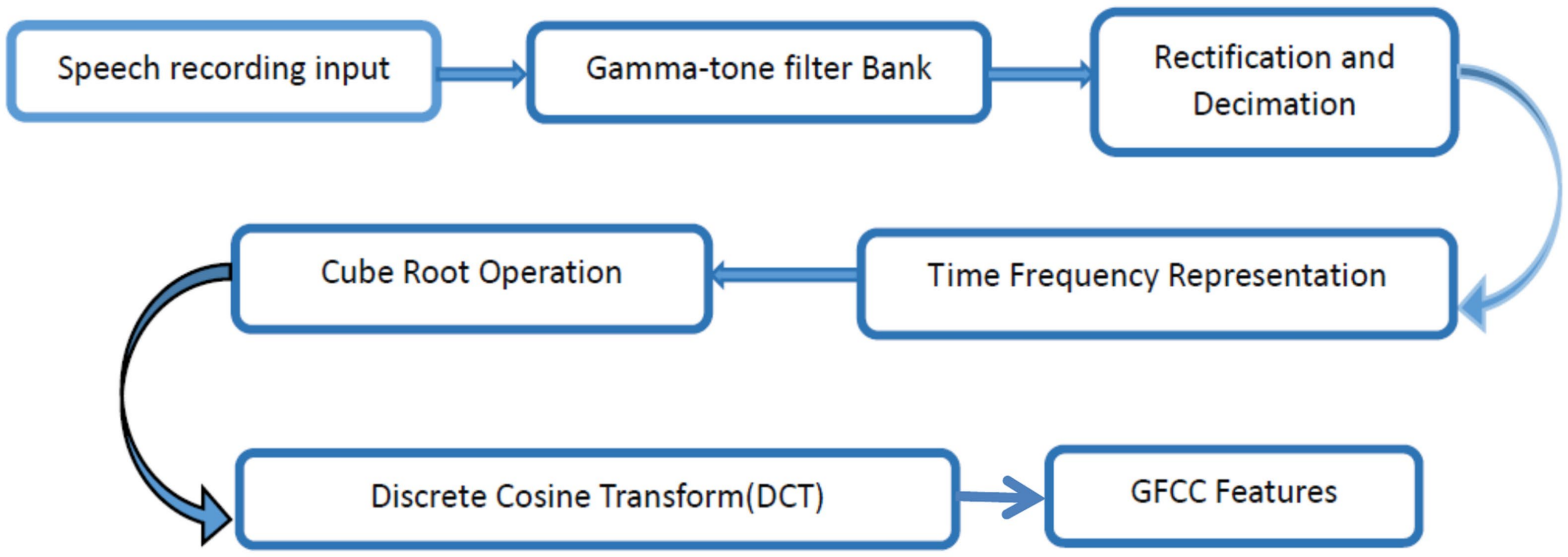
Steps to obtain GFCC.

Figure 2 shows the GFCC based feature extraction process. In extraction of GFCC, first the input signal is processed through a 64 channel filter bank that simulates cochlear filtering, converting the signal into the time-frequency domain. Then the filter responses are rectified and decimated to 100 Hz for time windowing. After that, the absolute values create a time-frequency representation, similar to a cochleagram. And the time-frequency representation undergoes a cube root transformation. Finally, the process concludes with the application of the discrete cosine transform (DCT). The filter output is shown in Equation 1.


$$g(t)=t^{n-1}e^{-2\mathrm{\pi bt}}\cos(2\pi f_{c}\;t)$$ (1)

Where t = time, n = filter order, b = bandwidth parameter, = Center frequency of the filter. The log compression can be defined as in Equation 2.


$$\log(E_{m})=\log(\int {|g(t)*x(t)|}^{2}\mathrm{dt})$$ (2)

Where = Gammatone filter function, =input audio signal, =energy in the m-th filter band. The discrete cosine transform (DCT) can be calculated as the Equation 3 given.


$$c_{k}=\sum_{m=1}^{M}\log(E_{m})\cos(\frac{\pi (m-0.5)k}{M})$$ (3)

Where = GFCC coefficient for the k-th band and M = Number of filter bands.

#### 2.2.2 Dwt

The term “wavelet” refers to a small finite-length function, which is used for analyzing non-stationary signals. Wavelets sampled at particular intervals are used in the discrete wavelet transform (DWT) method. By applying a “mother wavelet” function through translation and dilation, DWT breaks down a signal into two sets of coefficients: approximation coefficients, which capture low-frequency components, and detail coefficients, which reflect high-frequency components (Daubechies, 1992). For speech signals, the low-frequency approximation components are generally more informative and play a more significant role in defining the overall structure of the signal compared to high-frequency details (Chui, 1992). In DWT, the Haar wavelet serves as a fundamental tool for decomposing data into different frequency components, making it highly effective for signal compression and analysis. Its simple rectangular filter structure allows for minimal memory usage and fast computation, which makes the Haar wavelet particularly suitable for applications requiring efficiency and real-time processing. The wavelet coefficients for a signal can be computed using Equation 4.


$$\text{DWTj},k=\sum_{n}x[n].\mathrm{\varphi j},k[n]$$ (4)

Where DWTj, k = wavelet coefficient at scale j and translation k, X[n] = Discrete input signal, Φj, k [n] = wavelet function at scale j and translation k. The Haar decomposes a signal into approximation and detail coefficients at each level. The scaling function *ϕ*(t) and the wavelet function *φ*(t) for the Haar wavelet are defined as shown in Equation 5.

$$\phi (t)=\{\begin{array}{c} 1,0\leq t<1\\ 0,\text{otherwise} \end{array}$$ (5)

For the discrete input signal X[n], the decomposition at each level can be calculated as low-pass (approximation coefficients) and high- pass (detail coefficients).

The coefficients have been used as a feature for speech impairment detection, and the details of feature extraction have been explained in the proposed method section.

#### 2.2.3 Shallow learning methods

Shallow learning, often referred to as shallow machine learning or traditional machine learning, utilizes algorithms characterized by a simpler architecture, usually consisting of just one layer for transformation and learning. These models tend to be more interpretable and depend on methods such as recall and memorization, resulting in knowledge that is often more passive and may diminish over time (Prinzi et al., 2024; Xu et al., 2021). The shallow learning methods used for speech impairment that has SVM and MLP. The SVM method relies on calculating the decision boundaries to decide the class. MLP has also been used for SLI detection that has an input layer, a hidden layer and an output layer. Detailed implementation of these three shallow learning methods for speech impairment detection has been discussed in the proposed method.

#### 2.2.4 Shallow ensemble learning

Ensemble shallow learning enhances accuracy and robustness by aggregating predictions from various models. Shallow ensemble learning methods used here are bagging, boosting, stacking, etc. Bagging mitigates variance and over-fitting by training models on distinct subsets of data, although it can be demanding in terms of resources. The bagging method is implemented with an ensemble of decision trees that is called random forest. The output of the RF method can be given as in Equation 6.

$$\phi (t)=\{\begin{array}{c} 1,0\leq t<0.5\\ -1,0.5\leq t<1\\ 0,\text{otherwise} \end{array}$$ (6)

Where Ŷ = final prediction, T = number of decision trees, ht.(x) = prediction from the t-th tree. Boosting, on the other hand, sequentially addresses errors by concentrating on challenging instances, but it can be vulnerable to noise. The output of the gradient boosting can be represented with Equation 7.


$$\hat{Y}=\sum_{t=1}^{T}\eta .h_{t}(x)$$ (7)

Where ɳ = learning rate, ht. (x) = weak learner at iteration t. The XGBoost can be represented with Equation 8.

Ω(h)=λ‖ω‖2 (8)

Where T = no of leaves of the tree, ω = output scores of the leaves, *ϒ* = controls the minimum loss.

Stacking combines predictions from different models using a meta- learner, which can improve performance but may lead to over-fitting if data is not handled properly. Each approach has its own strengths and weaknesses, enabling ensemble learning to deliver more dependable predictions (Xu et al., 2021).

#### 2.2.5 Deep learning

**Figure 3:**
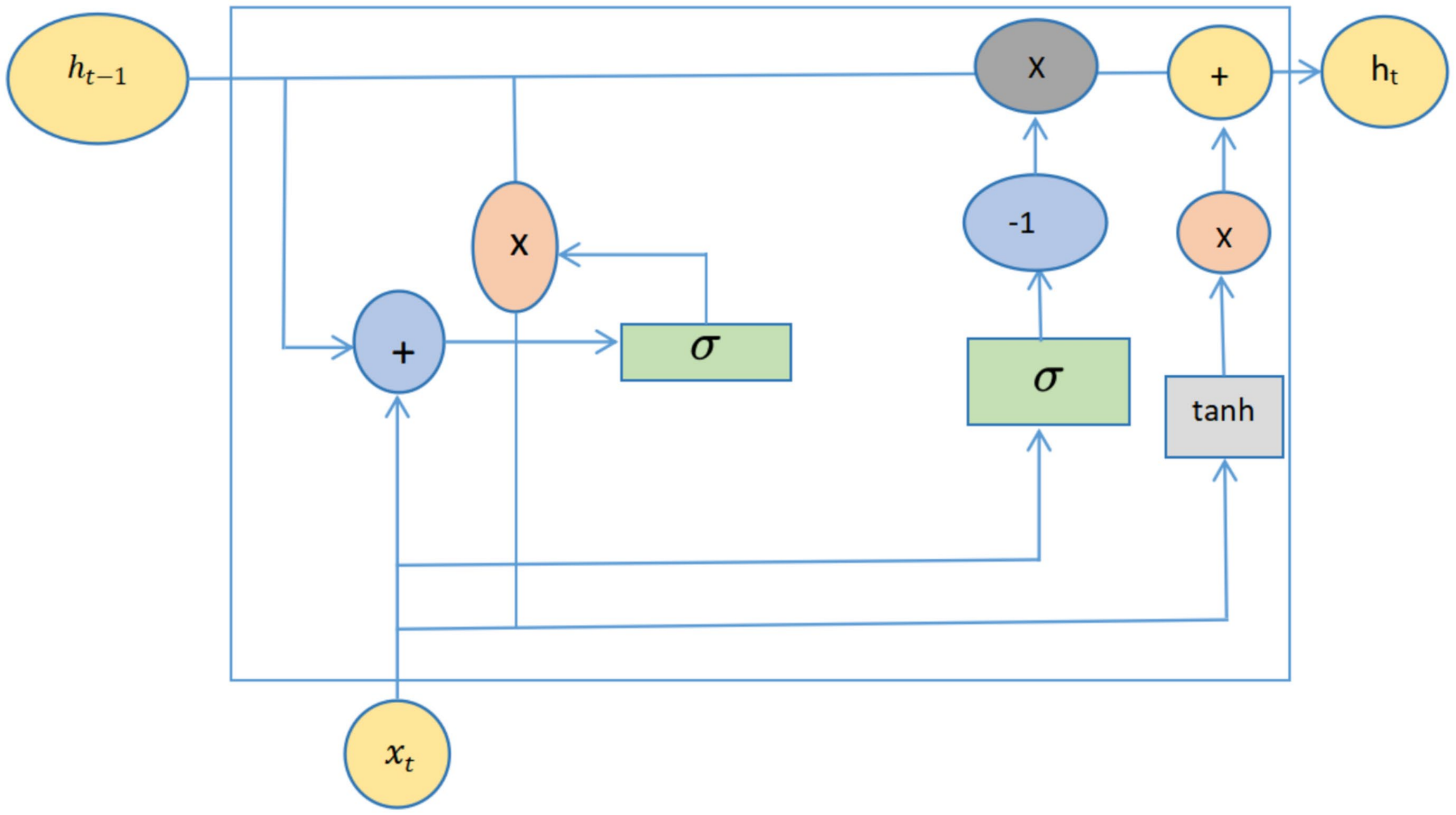
GRU architecture.

Deep learning (DL) is a transformative technique in artificial intelligence that utilizes multi layer neural networks to analyze vast amounts of data, revealing complex patterns within high dimensional datasets (Goodfellow et al., 2016). DL methods that are commonly used are CNN, LSTM, GRU, deep belief networks (DBN), and a combination of different networks. One of the earliest and most commonly used DL methods is CNN. The basic CNN architecture consists of an input layer, a filter layer, a convolutional layer, a pooling layer, and an output layer. The first layer of CNN is convolutional layer that extracts features from input layer and after filtering with the help of kernel. Pooling layers are used for reducing the dimension of the CNN. Max-pooling layer is widely used because of its high performance, as it picks the maximum values from the index and avoids the rest of the values. Another DL network that is used most commonly is LSTM. LSTM consists mostly of various types of gates to predict the output (Hochreiter and Schmidhuber, 1997). GRU is an RNN-based DL that can be trained in a short time and give improved training accuracy. The architecture of GRU with all the gates is shown in Figure 3.

The GRU has two gates: update gate and reset. Update gate decides the amount of past information (h t-1) to retain and the new information (Xt) to include. It helps capture long-term dependencies by controlling how much previous memory is passed to the current state. The update gate can be defined as in Equation 9.


Zt=σ(Wzxt+Urht−1+bz) (9)

Where Zt = Update gate value, Xt = Current input, ht-1 = Previous hidden state. Reset gate decides how much past information to forget. A value close to 0 indicates past memory, while a value close to 1 retains it. Hidden state (h t) combines information from the input (Xt) and previous state (h t-1) using gates. The reset gate can be defined as in Equation 10.


Rt=σ(Wrxt+Urht−1+br) (10)

Where Rt = reset gate value. The hidden state can be defined as in Equation 11.


ht=Zt⊙ht−1+(1−zt)⊙tanh(Whxt+Uh(rt⊙ht−1)+bh) (11)

Where ht = current hidden state, = element-wise multiplication. The information flow is scaled by an activation function to balance short-term and long-term dependencies. By using these gates effectively, GRU balances the retention of useful past information with the integration of new input for dynamic time-series tasks. Various combinations of DL methods are also used here for speech impairment detection, such as ConvLSTMs, BiLSTMs, CNN-LSTM and GRU-CNN. Detailed implementation of all the methods for speech impairment detection are discussed in the next section.

### 2.3 Feature extraction

**Figure 4:**
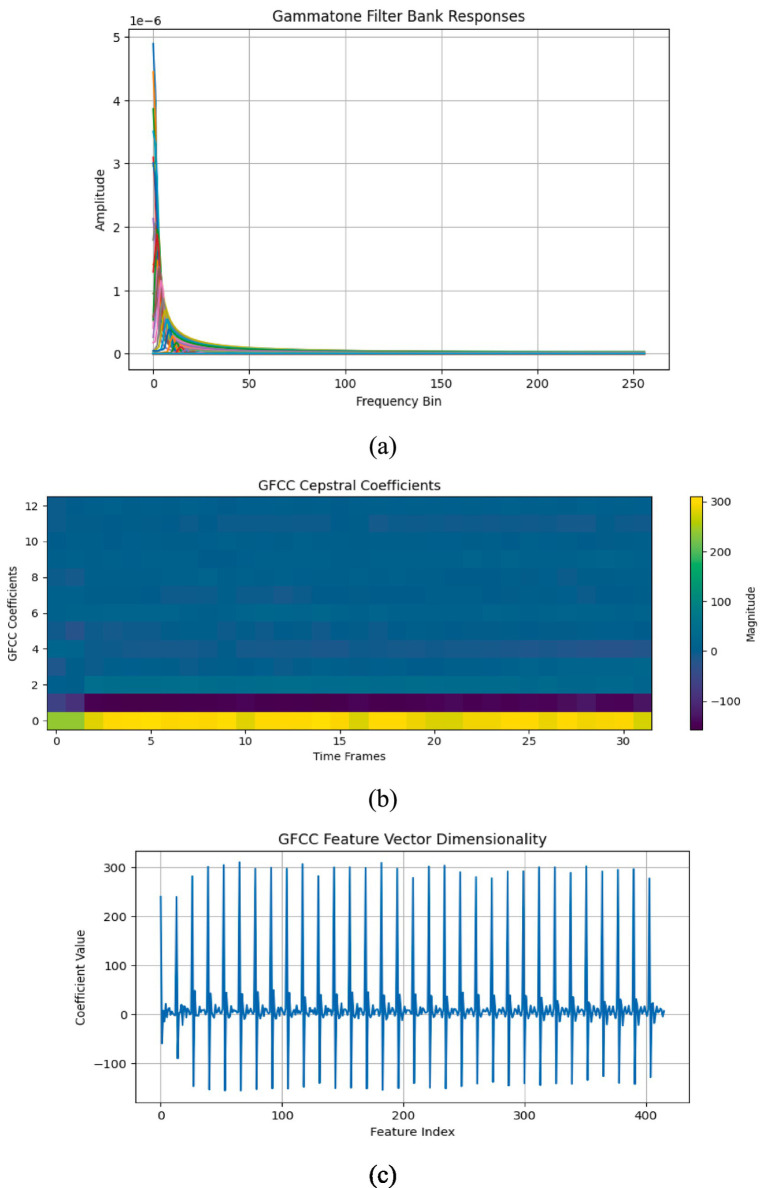
GFCC feature extraction: **(a)** gammatone filter bank response, **(b)** GFCC cepstral coefficients, **(c)** GFCC feature vector dimensionality.

Features extraction has been performed on the original signals of both healthy and patient individuals using both GFCC and DWT. The GFCC feature extraction visualization showing filter bank responses, cepstral coefficients, and feature dimensionality is shown in Figure 4.

**Figure 5:**
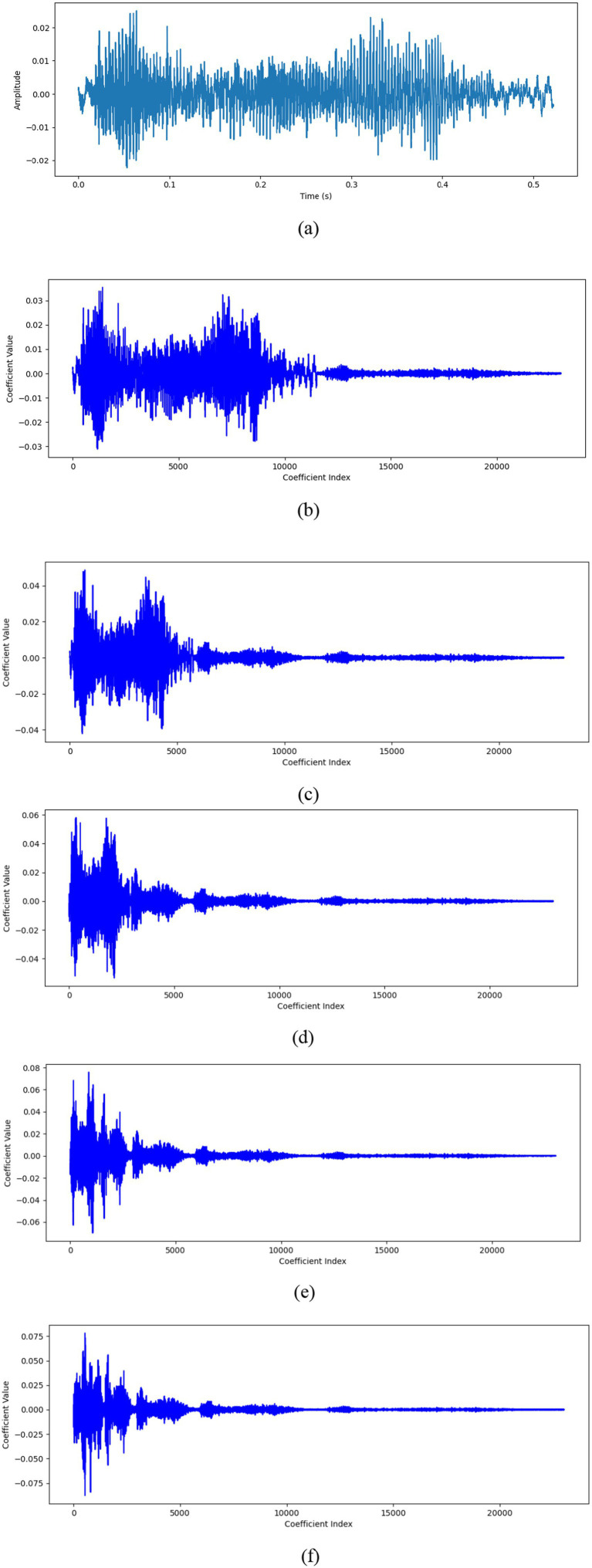
**(a)** Patients signal, **(b)** approximate coefficient at level 1, **(c)** approximate coefficient at level 2, **(d)** approximate coefficient at level, **(e)** approximate coefficient at level 4, **(f)** approximate coefficient at level 5.

A wavelet-based feature extraction method is employed to analyse speech signals and prepare features for classification. To enhance the extraction of relevant features from these signals, DWT is applied to extract time-frequency information. The procedure involves loading each speech signal, performing wavelet decomposition, and organizing the extracted features for machine learning analysis. For every speech sample, the DWT is applied using the Haar wavelet at a decomposition level of 5. A collection of wavelet coefficients representing the signal over several frequency bands is produced by this procedure. A patient speech signal’s time-domain waveform is shown in Figure 5A, and the approximation coefficients at different decomposition levels are shown in Figures 5B–5F. The wavelet coefficients indicate important areas where the patient signal deviates and show distinct changes in the energy distribution and signal patterns between healthy and patient recordings. By capturing the time frequency characteristic from the audio analysis this method enables the use of wavelet-transformed features into machine learning models.

### 2.4 Speech impairment detection method

The evaluation of the proposed model was performed using 5-fold cross-validation at the recording level. In this protocol, individual speech recordings were randomly divided into training and testing folds without enforcing speaker-level separation. As multiple recordings were obtained from each child, it is possible that recordings from the same subject appear in both training and testing sets. Therefore, the reported results reflect recording-level classification performance rather than strictly speaker-independent generalization. The extracted wavelet coefficients are utilized as input for training different models where each speech signals label of ‘0’ is for healthy and ‘1’ is patient. The hyper-parameter training method used is grid search method that evaluates all possible combinations of a specified set of hyper-parameters. To detect the speech impairments, a comprehensive approach is used with a range of techniques from shallow to deep learning according as referred from Table. 2.

**Table 2:** Parameters used for training.

Method used	Algorithm used	With GFCC feature		With DWT features	
		Parameters used	Training accuracy (%)	Parameters used	Training accuracy (%)
Shallow Learning	SVM	Kernel: Linear/Polynomial/RBF	-	Kernel: Linear/Polynomial/RBF	100.00
	MLP	Epochs-50, Batch size-32, Optimiser-Adam, Hidden Layer-2, Activation-Softmax	89.14	Epochs: 50, Batch size: 32, Optimiser: Adam, Hidden Layer 2, Activation: Softmax	100.00
Ensemble Shallow Learning	RF	n_estimators: 100	100.0	n_estimators: 100	100.00
	GBoosting	n_estimators: 100, Learning_rate: 0.1	85.81	n_estimators: 100, Learning_rate: 0.1	100.00
	XGBoost	n_estimators: 100, Learning rate: 0.1, random_state = 42	87.90	n_estimators: 100, Learning rate: 0.1, random_state = 42,	100.00
	CatBoost	Iterations: 1000, Learning Rate: 0.1, Depth: 6	90.47	Iterations: 1000, Learning Rate: 0.1, Depth: 6	100.00
	Light GBM	Number of Leaves: 31, Learning Rate: 0.1, n_estimators: 1000	92.97	Number of Leaves: 31, Learning Rate: 0.1, n_estimators: 1000	100.00
	Stacking	Base model: RF, SVC, GBoosting, XGBoost, Final estimator: LR	99.82	Base model: RF, SVC, GBoosting, XGBoost, Final estimator: LR	100.00
Deep Learning	1D Conv	Conv Layer1: 32, Conv Layer2: 64, Dense Layer: 128, epochs = 50, batch_size = 32, Optimizer: Adam	87.67	Conv Layer1: 32, Conv Layer1: 64, Dense Layer: 128, epochs = 50, batch_size = 32, Optimizer: Adam	100.00
	ConvLSTM	Optimizer: Adam, Learning Rate: 0.001, Epochs: 50, Batch Size: 64, Optimizer: Adam	89.21	Optimizer: Adam, Learning Rate: 0.001, Epochs: 50, Batch Size: 64, Optimizer: Adam	99.10
	BidirectionalLSTM	Epochs = 50, batch_size = 64, validation_split = 0.2, Optimizer: Adam, LSTM: 64	89.00	Epochs = 50, batch_size = 64, Optimizer: Adam, LSTM: 64	89.00
	DBN	BernoulliRBM(n_components = 64, learning_rate = 0.05, n_iter = 10)	80.00	BernoulliRBM(n_components = 64, learning_rate = 0.05, n_iter = 10)	97.00
	CNN- LSTM	Filters = 64, activation = relu, pool size = 2 for maxpooling, units = 50 for LSTM, optimizer = adam, Dense = 100, Activation = softmax, epochs = 50, batch_size = 32	87.39	Filters = 64, activation = relu, units = 50 for LSTM, optimizer = adam, Dense = 100, Activation = softmax, epochs = 50, batch_size = 32	100.00
	GRU-CNN	Conv1D(filters = 64, kernel_size = 3, activation = ‘relu’), Bidirectional(GRU(64, return_sequences = True)), Bidirectional(GRU(32)), epochs = 50, batch_size = 32	89.67	Conv1D(filters = 64, kernel_size = 3, activation = ‘relu’), Bidirectional(GRU(64)), Bidirectional(GRU(32)), epochs = 50, batch_size = 32	100.00

**Figure 6:**
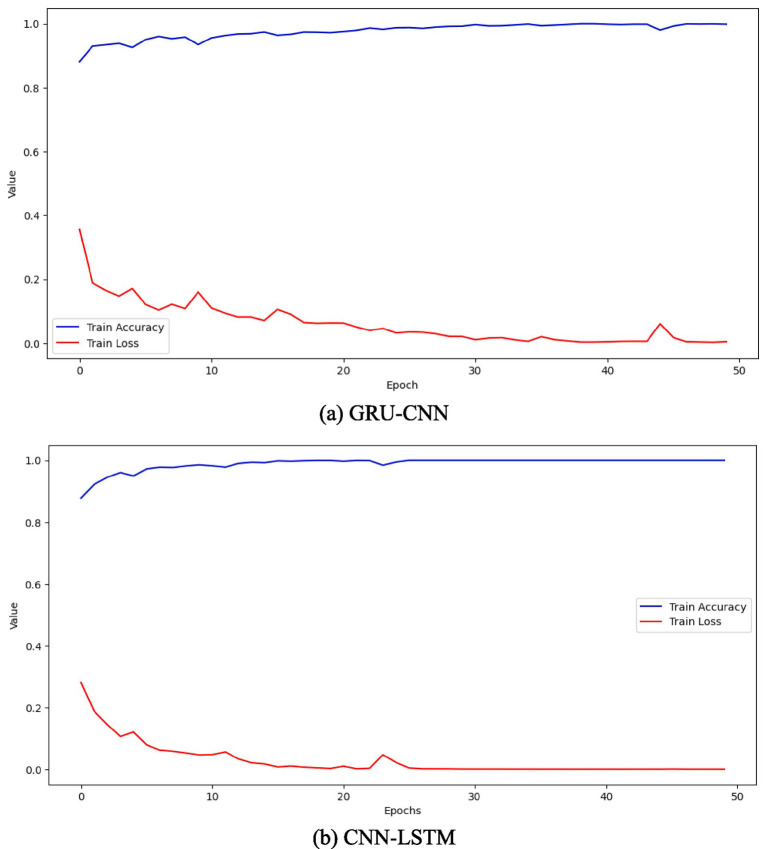
Training performance with DWT features: **(a)** GRU-CNN, **(b)** CNN-LSTM.

Shallow learning achieves nearer to 100% with DWT features and given vary results with GFCC features. Similarly, ensemble method such as RF, GBoost, XGBoost, CatBoost and LightGBM achieve 100% accuracy with DWT. Deep learning methodologies, including 1D Conv, ConvLSTMs, BiLSTMs, DBN, CNN-LSTM, GRU-CNN employ multiple layers of interconnected neurons to discover detailed patterns and representations from inputs. 1D Conv are composed of two convolutional layers followed by a dense layer, achieving an accuracy of 87.67% with GFCC and 100% with DWT. ConvLSTM reaches accuracy of 89.21% with GFCC and 99.10% with DWT. BiLSTM demonstrates consistent performance, attaining 89% accuracy for both feature types. DBN are trained with 64 components, achieving 80% accuracy with GFCC and 97% with DWT. CNN-LSTM and GRU-CNN has achieved an accuracy of 100% with DWT features. Figure 6 shows the training accuracy and training loss of the CNN-LSTM and GRU-CNN methods.

## 3 Results

All experiments were implemented in Python using Google Colab environment. Feature extraction was performed using librosa and pywt libraries. DWT features were computed using Haar wavelet with decomposition level 5. Machine learning models were implemented using scikit-learn and TensorFlow libraries. Feature normalization was performed using StandardScaler. Random seed value of 42 was used where applicable to ensure reproducibility. The presented approach has been tested and executed using python in Google Colab in i7 processor with 32GB RAM. The results of all model evaluations using various learning methods and feature extraction techniques has been represented here specially focusing on GFCC and DWT. This analysis highlights the executions of different methods as to precision, recall, F-score, accuracy and AUC score.

Where TP = True Positives, FP = False Positives, FN = False Negatives.

### 3.1 Analysis of accuracy

**Figure 7:**
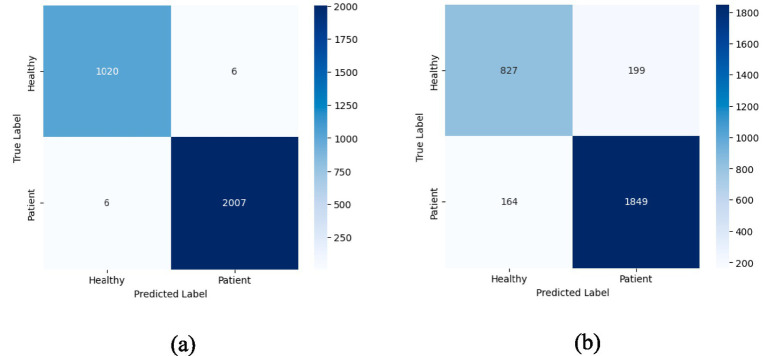
Confusion matrix of GRU-CNN method: **(a)** DWT feature and **(b)** GFCC feature.

Accuracy represents the proportion of accurate predictions to the total prediction made. The confusion matrix of GRU-CNN method with DWT features and GFCC feature is shown in Figure 7. Table 3 shows the percentage accuracy of different methods used. Boosting methods and 1D Conv achieved particularly high accuracy with wavelet features, reflecting their ability to handle the complex patterns that these features capture. GRU-CNN method achieves 99.67% accuracy that is highest among all methods. The accuracy suggests that the speech impairment detection method can be used effectively for monitoring.

**Table 3:** Testing accuracy of different methods with different features.

Method used	Method used	GFCC	DWT
Shallow Learning	SVM	–	94.41%
	MLP	88.72%	91.61%
Ensemble Shallow Learning	RF	88.28%	94.57%
	GBoost	85.80%	95.39%
	XGBoost	87.24%	94.57%
	CatBoost	88.41%	95.89%
	Light GBM	88.13%	95.89%
	Stacking	88.83%	95.89%
Deep Learning	1D Conv	87.12%	98.85%
	BiLSTM	88.59%	89.47%
	ConvLSTM	88.65%	94.08%
	DBN	80.11%	89.91%
	CNN- LSTM	86.95%	98.36%
	GRU- CNN	87.37%	99.67%

The confusion matrix represents classification results obtained from one evaluation subset generated during the validation process. Due to preprocessing filtering and validation splitting used in deep learning experiments, the total number of samples appearing in the confusion matrix may differ slightly from the total number of recordings available in the dataset. Reported performance metrics represent averaged values obtained across repeated experiments.

### 3.2 Analysis of precision, recall and F-score

For high-precision voice classification applications, DWT-based features show great applicability, especially when paired with sophisticated machine learning methods. The recall ratings for every approach for both feature sets are shown in Table 4. The GRU-CNN model’s architecture successfully captures the local and time-dependent patterns stored in the wavelet domain, as evidenced by its perfect recall of 1.00 when employing DWT features. Deep hybrid architectures and DWT features are compatible, as evidenced by this great sensitivity to positive cases. The findings demonstrate the efficiency of wavelet-based features, particularly when combined with deep learning and boosting techniques, for accurate speech impairment detection.

**Table 4:** Performance of different methods.

Method used	Method used	Precision		Recall		F-score		AUC	
		GFCC	DWT	GFCC	DWT	GFCC	DWT	GFCC	DWT
Shallow Learning	SVM	–	0.94	–	0.94	–	0.94	-	0.97
	MLP	0.89	0.92	0.89	0.92	0.88	0.92	0.94	0.95
Ensemble Shallow Learning	RF	0.89	0.97	0.96	0.95	0.92	0.96	0.93	0.99
	GBoost	0.86	0.95	0.95	0.98	0.91	0.97	0.89	0.99
	XGBoost	0.88	0.95	0.96	0.97	0.88	0.95	0.92	0.99
	CatBoost	0.89	0.97	0.95	0.97	0.92	0.97	0.93	0.99
	Light GBM	0.89	0.97	0.95	0.97	0.92	0.97	0.92	0.99
	Stacking	0.88	0.96	0.88	0.96	0.88	0.96	0.93	0.99
Deep Learning	1D Conv	0.87	0.99	0.87	0.99	0.87	0.99	0.92	1.00
	BiLSTM	0.88	0.90	0.89	0.89	0.88	0.89	0.94	0.94
	ConvLSTM	0.89	0.94	0.89	0.94	0.88	0.94	0.93	0.99
	DBN	0.81	0.90	0.93	0.94	0.87	0.92	0.81	0.96
	CNN-LSTM	0.87	0.98	0.87	0.98	0.87	0.98	0.92	1.00
	GRU- CNN	0.89	1.00	0.94	1.00	0.91	1.00	0.99	1.00

### 3.3 Analysis of AUC

**Figure 8:**
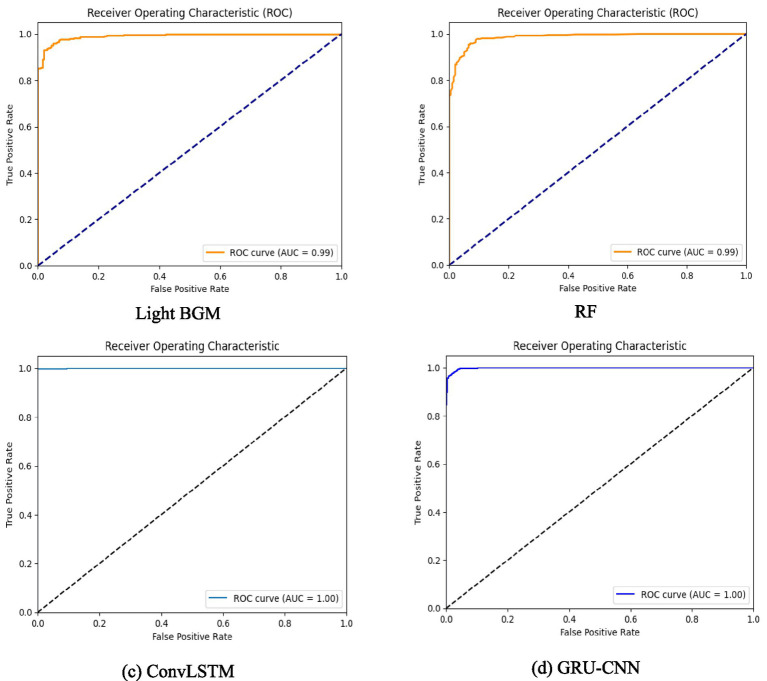
ROC of different methods: **(a)** Light BGM, **(b)** RF, **(c)** ConvLSTM, **(d)** GRU_CNN.

A crucial metric for assessing binary classification models is the Area Under the Curve (AUC) of the Receiver Operating Characteristic (ROC) curve, especially when dealing with imbalanced datasets. The AUC values for several shallow and deep learning techniques are shown in Table 4. As shown in Figure 8, the AUC findings unequivocally show that wavelet-based features are superior to GFCC features across several models. Overall, the analysis highlights that wavelet features are highly effective for speech classification tasks, offering enhanced representation of both time and frequency domain characteristics.

## 4 Discussion

A detailed analysis of detection methods using shallow learning, ensemble learning and deep learning are compared. The comparison of implemented methods and complexity of the existing methods are discussed below.

### 4.1 Comparison of all implemented methods

The findings show that the model architecture and feature extraction technique used have a significant influence on classification accuracy. Specifically, discrete wavelet transform (DWT) shows up as a very successful feature extraction method that significantly outperforms GFCC. The two top-performing models for each feature type consistently demonstrate that DWT offers a more informative and discriminative representation of speech signals, as shown in Table 5. All of the results show that DWT-driven deep learning techniques present a strong option for precise and trustworthy speech impairment identification.

**Table 5:** Comparison of different implemented methods.

Matrices	Method used	GFCC	DWT
Precision	GRU- CNN	0.89	1.00
	1D Conv	0.87	0.99
Recall	GRU- CNN	0.94	1.00
	1D Conv	0.87	0.99
F-score	GRU- CNN	0.91	1.00
	1D Conv	0.87	0.99
AUC	GRU- CNN	0.92	1.00
	CNN- LSTM	0.92	1.00
	1D Conv	0.92	1.00
	ConvLSTM	0.93	0.99
	RF	0.93	0.99
	GBoost	0.89	0.99
	XGBoost	0.92	0.99
	CatBoost	0.93	0.99
	Light GBM	0.92	0.99
	Stacking	0.93	0.99
Accuracy	GRU- CNN	87.37%	99.67%
	1D Conv	87.12%	98.85%
	CNN- LSTM	86.95%	98.36%

### 4.2 Complexity of GRU-CNN method

In order to ensure computational efficiency, the suggested Bidirectional GRU-CNN architecture is specifically designed to describe both long-range temporal dependencies and localized patterns within speech signals. The network starts with a one-dimensional convolutional layer using the ReLU activation function and 64 filters of kernel size 3. This layer extracts important local information from the wavelet-transformed speech inputs and adds non-linearity. The feature maps are then compressed via a max-pooling layer to lower dimensionality and processing burden. A dropout layer with a rate of 0.3 is used to stop over fitting. Two bidirectional GRU layers with 64 and 32 units, respectively, follow the convolutional block. These layers enhance the learned representation by capturing temporal dependencies throughout the signal’s whole time range by processing the sequential input in both forward and reverse orientations. To improve generality even more, a dropout layer is added after the GRU stack. The model ends with a dense layer that uses a sigmoid activation function to create a binary classification result that indicates whether or not speech impairment is present.

Overall, the model consists of approximately 136,000 trainable parameters, which strikes a balance between learning capacity and over-fitting risk. With only a single convolutional layer and two GRU layers, the architecture remains relatively lightweight compared to deeper neural networks, enabling fast training and low memory consumption. Simulation was performed on a system with an i7 processor and 32GB of RAM using the Google Colab platform. Each epoch completed in approximately 1–2 min, demonstrating the model’s practicality for real-time or clinical deployment. Cross-validation has been done for GRU-CNN method with DWT features. The results obtained have accuracy of 0.9947 ± 0.0024, precision of 0.9955 ± 0.0018, recall of 0.9965 ± 0.0020, F1 Score of 0.9960 ± 0.0018 and AUC of 0.9998 ± 0.0001. The results obtained for cross validation method is nearly same as the result obtained with the train test split methods. Hence, there is no model parameter uncertainty that will happen.

### 4.3 Comparison with MFCC features

The proposed method has been compared with MFCC features as shown in Table 6. The precision, recall, F-score and AUC are higher for DWT features. It can be observed from Table 6 that the DWT features give better performance in detecting the speech impairment than the MFCC features.

**Table 6:** Comparison with MFCC features.

Method used	Precision		Recall		F-score		AUC	
	MFCC	DWT	MFCC	DWT	MFCC	DWT	MFCC	DWT
1D Conv	0.99	0.99	0.99	0.99	0.99	0.99	0.99	1.00
ConvLSTM	0.99	0.94	0.99	0.94	0.99	0.94	0.99	0.99
DBN	0.72	0.90	0.99	0.94	0.83	0.92	0.60	0.96
GRU- CNN	0.99	1.00	0.99	1.00	0.99	1.00	0.99	1.00

### 4.4 Validation with other data-set

**Figure 9:**
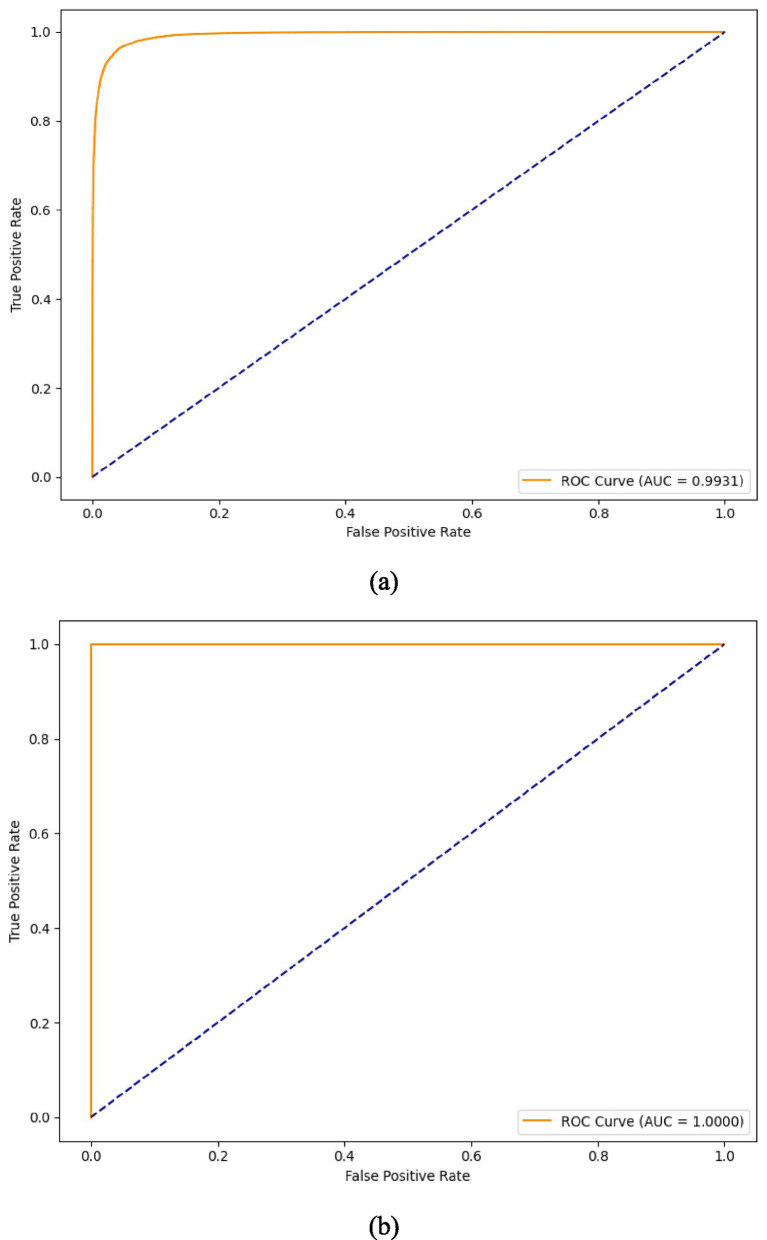
ROC-AUC of GRU-CNN method with **(a)** GFCC features and **(b)** DWT features.

The presented GRU-CNN has been tested with TORGO data-set (Rudzicz et al., 2012). The GRU-CNN technique has been tested with different features and the outcomes are given in Table 7. It has a maximum accuracy of 100% with DWT features. Figure 9 shows the AUC of the GRU-CNN method with GFCC features and DWT features. Precision, recall and F-score of DWT features are higher than GFCC features. Hence, it can be concluded that the intended approach can be implemented for speech impairment observation effectively.

**Table 7:** Performance with TORGO data-set.

Features used	Precision	Recall	F-score	AUC	Accuracy
GFCC	0.952	0.967	0.959	0.993	95.94%
DWT	1.00	1.00	1.00	1.00	100.0%

### 4.5 Comparison with existing method

The analysis of various techniques and feature extraction methods applied to the LANNA dataset, emphasizing the uniqueness and benefits of the proposed approach is shown in Table 8; Lipkin (2022).

**Table 8:** Comparison with other methods for LANNA data-set.

Authors	Features used	Techniques used	Performance	Computational efficiency	Model complexity	Memory requirements	Observation
Sharma and Singh (2020b)	NCA features	Weighted k-NN classifier	Accuracy - 97.93%	High (3 optimized NCA features are used)	Low (k-NN with small feature set)	Low	Only vowel ‘a’ is tested.
Sharma and Singh (2020a)	Linear Predictive Coefficients (LPC)	Naive Bayes	Accuracy - 97.9% (top 20 LPC) Accuracy - 97.8 (top 10 LPC)	Moderate (Due to large feature set)	Moderate (Feature selection + Classifier)	Moderate	Only vowel ‘a’ is tested.
Sharma G et al. (2020)	Local binary patterns	SVMs k-NN Complex Tree	Accuracy - 97.36%	Moderate (Multiple feature extraction and classifier used)	High (Due to multiple classifier and feature fusion)	High (107 dimensional feature vector)	Combined speech module is not tested.
Sharma and Singh (2020b)	CNN	Hybrid CNN-LSTM model	Accuracy −100% Reliability indexes over 90%.	Moderate (Due to deep learning training)	High (Multiple CNN layers and LSTM)	High (Due to deep neural network layers)	Only vowel speech is used. Consonant, syllable and difficult word speech not tested. Combined speech module is not tested.
Barua et al. (2023)	Favipiravir pattern, statistical feature extractor, and wavelet packet decomposition	SVM classifier	Accuracy 99.87% (tenfold), 98.80% (LOSO)	Moderate (Due to multi stage feature extraction)	Moderate (feature extraction and SVM)	Moderate	Only vowels tested. Consonant, syllable and difficult word speech not tested. Combined speech module is not tested.
Reddy et al. (2020)	Glottal features and MFCC	Feed-forward Neural Network	Accuracy - 98.82%	Moderate (Due to multiple feature extraction processes)	Moderate (Feature extraction and Neural classifier)	Moderate (Due to high dimensional vectors)	Vowels, single syllable, bi-syllabic words, tri-syllabic words, four syllable and difficult word not tested. Combined speech module is not tested.
Kasture and Jain (2024)	Combination of MFCC and i-vectors	MobileNet-v2	Accuracy - 98.70%	Moderate (Due to deep learning models training)	High	High	Vowels, consonant and syllable individually used. Combined speech module is not tested.
Slogrove and van der Haar (2020)	MFCC	Random forest	Accuracy - 99%	High (Due to classical ML models)	Low	Low	Accuracy decreases for more than three syllables. Combined speech module is not tested.
Proposed Method	DWT features	GRU-CNN	Accuracy - 99.67%	Moderate (efficient training with lightweight architecture)	Modera	Moderate (model contain only one CNN layer and two GRU layers)	All speech recordings in a single module is used.

As summarized in Table 8, previous research has explored different methodologies, achieving impressive performance but also facing significant limitations. To justify the contribution of this work, a comprehensive comparison including computational efficiency (training/inference time), model complexity (number of parameters), memory requirements, and clinical deployability has been added. A hybrid CNN-LSTM model that was limited to using vowel speech only and did not include consonants, syllables, or challenging words has been utilized (Sharma G. et al., 2021). Local binary pattern features with SVM, k-NN, and complex tree classifiers have been applied, achieving an accuracy of 97.36% (Sharma G. et al., 2021); however, similar to several earlier works, their study did not evaluate a unified or combined speech module. Although these investigations were limited to certain speech kinds, other noteworthy contributions are reported having accuracies of 98.82% (Reddy et al., 2020) and 98.70% (Kasture and Jain, 2024), respectively. Statistical feature extraction is used to achieve an exceptional accuracy of 99.87% (Barua et al., 2023), however their study was limited to vowel-based speech. Similar to this, MFCC features has been used with a random forest classifier, but their work lacked a thorough speech module and they saw a drop in performance for speech inputs longer than three syllables (Slogrove and van der Haar, 2020). Using NCA and LPC characteristics, also reported competitive results (Sharma and Singh, 2020a, 2020b); however, their trials were limited to the vowel “a.” Some of the existing methods are more accurate but they are also more complex and require more memory. The proposed method is less complex, and more accurate than existing work.

The complementing capabilities of the two architectural components of the proposed GRU-CNN model are responsible for its remarkable performance. When examining speech deficits that occur over whole sequences, GRU layers are naturally good at simulating long-term temporal linkages. The CNN layers, on the other hand, are skilled at extracting discriminative, localized patterns from wavelet-transformed inputs that preserve rich information in both the time and frequency domains. The DWT greatly improves this procedure. DWT is especially well-suited for pathology-oriented speech tasks because it offers a multi-resolution analysis that captures minute variations in speech structure, in contrast to MFCC, which may lose fine-grained frequency information. This results in consistently strong performance outcomes, including a precision score of 1.00, recall of 1.00, F1-score of 1.00, and an overall accuracy of 99.67%, all achieved with minimal indications of over-fitting.

The advantages of the proposed method can be outlined as follows:

- The proposed GRU-CNN method employs an innovative hybrid architecture that achieves an impressive accuracy of 99.67%.
- The proposed method assesses the entire data-set, including vowels, consonants, syllables, and challenging words.
- The proposed method uses time frequency features of speech signal.
- It uses vowels, consonants, syllables, and challenging words in one module to identify speech impairment while existing methods have designed separate modules for each type of speech.
- DWT features offer a simultaneous localization in time and frequency domain that helps in increasing accuracy more than only frequency domain features.

### 4.6 Limitations

A limitation associated with this study is that cross-validation was carried out using recordings rather than subjects. As multiple recordings were made for each child, there could be cases where recordings belonging to one subject exist in both training and test datasets. This may enable the models to learn certain features related to speakers independently of speech impairments. However, the outcomes of the study suggest that the extracted time-frequency features have excellent discriminatory capabilities. Further research needs to be conducted to verify the generalization performance of the model on other datasets.

### 4.7 Ethics statement

The current research relies on a publicly available dataset that has been created by LANNA, Czech Technical University in Prague, in conjunction with Motol University Hospital and Charles University. It consists of audio recordings from children with speech impairment and those without it. All the recordings have been made with the consent of the relevant institutional ethics committee and de-identified before publication. For this reason, no further ethical approval is required for the current study since it involves secondary data.

## 5 Conclusion

A method for detecting speech impairment has been introduced by using GRU-CNN deep learning and time frequency characteristics of speech. This proposed method highlights the potential of identifying children’s speech difficulties. The DWT and hybrid deep learning bidirectional GRU-CNN method demonstrated remarkable 99.7% accuracy result, which indicates that these techniques could help clinicians and speech therapists by providing quick and better evaluations of speech impairments. Although these results are promising, further validation on larger clinical datasets is necessary to improve generalizability and clinical reliability. The limitation of the method is that its performance may reduce if the signals are not recorded properly. Another limitation of the work is that GRU-CNN is a complex network. In the future, a less complex model will be implemented. Future research should aim to validate these findings using a wider range of datasets to confirm that the models can generalize effectively across various speech characteristics and demographic groups. Furthermore, investigating different wavelet based feature extraction methods could yield additional insights, and the integration of more advanced hybrid architectures might give new opportunities to enhance in performance. In this experiment, however, the evaluations were carried out based on the recordings. Future investigations to examine the generalization of the methods should carry out a speaker independent evaluation of the system.

## Data Availability

The original contributions presented in the study are included in the article/supplementary material, further inquiries can be directed to the corresponding author.

## References

[ref1] Arora S. Neeru N. (2016). Speech identification using GFCC, additive white gaussian noise (AWGN) and wavelet filter. Int. J. Comput. Appl. 146, 17–24. doi: 10.5120/ijca2016910854

[ref3] Barua P. Aydemir E. Dogan S. Erten M. Kaysi F. Tuncer T. . (2023). Novel favipiravir pattern-based learning model for automated detection of specific language impairment disorder using vowels. Neural Comput. & Applic. 35, 6065–6077. doi: 10.1007/s00521-022-07999-4, 36408288 PMC9660223

[ref4] Chui C. K. (1992). An Introduction to Wavelets. New York, NY: Academic Press, Inc.

[ref5] Danescu C.V. Enescu F.m. Bizon N. Sebe Ș.A. Selea M. (2025). “Designing a smart device for personal assistance based on artificial intelligence,” in *International Conference on Electronics, Computers and Artificial Intelligence (ECAI)*, 1–10.

[ref6] Daubechies I. (1992). Ten Lectures on Wavelets. Philadelphia, PA: Society for Industrial and Applied Mathematics.

[ref7] Deepa N. (2023). “Children specifically language impairment severity level prediction using improved conditional random fields and comparison with traditional models,” in *3rd International Conference on Innovative Practices in Technology and Management (ICIPTM)*, Uttar Pradesh, India, 1–6.

[ref8] Doru C. Costel B. (2023). “Classification of image classes based on the PCA algorithm optimized by the KNN algorithm improved by genetic algorithms,” in *International Conference on Information Communication and Signal Processing (ICICSP)*, 103–108.

[ref9] Dutta A. K. Wahab Sait A. R. (2024). A fine-tuned CatBoost-based speech disorder detection model. JDR. 3:27. doi: 10.57197/JDR-2024-0027

[ref10] Fu J. Yang S. He F. He L. Li Y. Zhang J. . (2021). Sch-net: a deep learning architecture for automatic detection of schizophrenia. Biomed. Eng. Online 20:75. doi: 10.1186/s12938-021-00915-2, 34344372 PMC8336375

[ref11] Goodfellow I. Bengio Y. Courville A. (2016). Deep Learning. New York, NY: The MIT Press.

[ref13] Hochreiter S. Schmidhuber J. (1997). Long short-term memory. Neural Comput. 9, 1735–1780. doi: 10.1162/neco.1997.9.8.1735, 9377276

[ref14] Ilie A. Manaila-Maximean D. Danila O. (2025). Efficient modeling of polarized reflection spectra in metasurfaces using deep learning frameworks. Univ. Polite Bucharest Sci. Bullet. Series Appl. Mathematics Phys. 87, 189–198. Available online at: https://www.scientificbulletin.upb.ro/static/pdfs/fullcf3_655397.pdf

[ref16] Kasture N. Jain P. (2024). Automatic recognition of disordered children’s speech signal in dyadic interaction using deep learning models. Multimed. Tools Appl. 83, 49493–49513. doi: 10.1007/s11042-023-17461-9

[ref17] Kaushik M. Baghel N. Burget R. Travieso C. Dutta M. K. (2021). SLINet: dysphasia detection in children using deep neural network. Biomed. Signal Process. Control 68:102798. doi: 10.1016/j.bspc.2021.102798

[ref18] Lipkin B. (2022). LANNA Dataset. Available online at: https://figshare.com/articles/dataset/New_draft_item/2360626?file=4000132

[ref19] Prinzi F. Currieri T. Gaglio S. Vitabile S. (2024). Shallow and deep learning classifiers in medical image analysis. Eur Radiol Exp. 8:26. doi: 10.1186/s41747-024-00428-2, 38438821 PMC10912073

[ref20] Reddy M. K. Alku P. Rao K. S. (2020). Detection of specific language impairment in children using glottal source features. IEEE Access 8, 15273–15279. doi: 10.1109/access.2020.2967224

[ref21] Remya M. S. Raman R. Sankaran R. Namboodiri V. Nedungadi P. (2025). Artificial intelligence for speech classification and enhancement of speech and language disorders: techniques, applications, and future directions. IEEE Access 13:114. doi: 10.1109/access.2025.3620114

[ref23] Rudzicz F. Namasivayam A. K. Wolff T. (2012). The TORGO database of acoustic and articulatory speech from speakers with dysarthria. Lang. Resour. Eval. 46, 523–541. doi: 10.1016/j.specom.2011.10.006

[ref24] Safdar S. Kausar S. Tehsin S. Mahmood M. Naif Alwakid G. (2023). “Prediction of specific language impairment in children using cepstral domain coefficients,” in *International Conference on Business Analytics for Technology and Security (ICBATS), Dubai, United Arab Emirates*, 1–11.

[ref25] Sharma G. Prasad D. Umapathy K. Krishnan S. (2020). “Screening and analysis of specific language impairment in young children by analyzing the textures of speech signal,” in *Annu Int Conf IEEE Eng Med Biol Soc.* 964–967. 10.1109/EMBC44109.2020.9176056 33018145

[ref26] Sharma Y. Singh B. (2020a). “Prediction of specific language impairment in children using speech linear predictive coding coefficients,” in *First International Conference on Power, Control and Computing Technologies (ICPC2T), Raipur, India*, 305–310.

[ref27] Sharma Y. Singh B. K. (2020b). “Classification of children with specific language impairment using pitch-based parameters,” in *IEEE Recent Advances in Intelligent Computational Systems (RAICS)*, Thiruvananthapuram, India, 42–46.

[ref28] Sharma Y. Singh B. K. (2021). One-dimensional convolutional neural network and hybrid deep-learning paradigm for classification of specific language impaired children using their speech. Comput. Methods Prog. Biomed. 213:106487. doi: 10.1016/j.cmpb.2021.106487, 34763173

[ref29] Sharma G. Zhang X. Umapathy K. Krishnan S. (2021). Audio texture and age-wise analysis of disordered speech in children having specific language impairment. Biomed. Signal Process. Control. 66:102471. doi: 10.1016/j.bspc.2021.102471

[ref30] Slogrove K. J. van der Haar D. (2020). “Specific language impairment detection through voice analysis,” in *23rd International Conference, BIS 2020*, 130–141.

[ref31] Spence S. H. (2003). Social skills training with children and young people: theory, evidence and practice. Child Adolesc. Mental Health 8, 84–96. doi: 10.1111/1475-3588.00051, 32797550

[ref32] Tomblin J. B. Records N. L. Buckwalter P. Zhang X. Smith E. O'Brien M. (1997). Prevalence of specific language impairment in kindergarten children. J. Speech Lang. Hear. Res. 40, 1245–1260. doi: 10.1044/jslhr.4006.1245, 9430746 PMC5075245

[ref33] Xu Y. Zhou Y. Sekula P. Ding L. (2021). Machine learning in construction: from shallow to deep learning. Dev. Built Environ. 6:100045. doi: 10.1016/j.dibe.2021.100045

[ref001] Zhang X. Lan B. Zhang L. (2025). Improving deep learning-based instance segmentation for crystallization trails with residual multi-scale feature and attention mechanisism. U. P. B. Sci. Bull. 87, 1. Available online at: https://www.scientificbulletin.upb.ro/static/pdfs/fullb35_925526.pdf

[ref34] Zhao D. Qiu Z. Jiang Y. Zhu X. Zhang X. Tao Z. (2024). A depthwise separable CNN-based interpretable feature extraction network for automatic pathological voice detection. Biomed. Signal Process. Control. 88:105624. doi: 10.1016/j.bspc.2023.105624

